# Malingering

**Published:** 1888-08-11

**Authors:** W. Curran

**Affiliations:** A.M.D.


### MALINGERING.

By W. CURRAN, A.M.D.

( Continued from p. 289. )

THE eye is a popular organ with your true malingerer, and all disturbances, circulatory or otherwise, should be viewed with suspiciou. Thus, a speck of tobacco juice, introduced under the lower lid a short time before the visit to the surgery will cause the unwary medical officer to run in vain through the whole gamut of collyria, whilst if tobacco is not forthcoming—as it is not as a rule in H.M. prisons—the wily prisoner will seek to throw dust in the eyes of the doctor by inserting a few grains into his own. Dr. Quintin, taking such a patient unawares on one occasion, removed three pieces of watch wire, two from under the upper, and one from under the lower lid. Surgeon Sibbald, R.N., tells how superior a morsel of tobacco leaf is to the speck of juice for this purpose—it can be removed before the surgeon's visit, and leaves no stain. Lime, too, is a popular irritant.

A good case of assumed blindness was exposed by Mr. Smalley. On taking a fresh appointment, he found a man in hospital suffering from discharge from both eyes, and supposed to be quite blind. The resistance he offered to all examination excited suspicion, so, after careful treatment for some few days, with a view to lessen the inflammation, chloroform was administered, and one cornea was found quite clear, whilst a slight opacity on the other was in such a position as could not interfere with vision. He was sent out of hospital and put to light labour, but would not give in. He refused to work, persisted he could not see, and ostentatiously tumbled over objects in his way when walking about. Reported for not working, he almost succeeded in pursuading the executive that he was being harshly treated; however, the surgeon carried the day, and he was punished. In a day or two he went to the governor and told him that the Lord had had mercy on him and had opened his eyes, so that he could see a little, and he felt sure if he was put to a certain class ofwork that he would soon see more.

Deafness is far more commonly assumed by soldiers than by prisoners, it being obvious that the former cannot conform with army discipline unless hearing is fairly good. Two good cases of shamming deafness have been given me by Surgeon Major Curran. In one, a man was sent from some up-country station to Calcutta, and in spite of suspicions, gained his discharge and was sent on board a homeward-bound vessel. Just as the vessel was about to be cast off, and in the excitement of bidding good bye to friends, a confederate of the doctor's wispered in his ear "Three cheers for old England!" a loyal sentiment to which the deaf man responded at once, to his cost.

The other case was still more extraodinary, as the subject was a sergeant of eighteen years' standing, and not at first sight a likely malingerer. He too was recommended for discharge, but was entrapped into an expression of joy by a whispered assurance that his papers had arrived.

In the prison service, although serious attempts at deceit are far from uncommon, it is chiefly with petty little complaints of imaginary ailments that prisoners seek to take in the medical officer. With this laudable end in view, various carefully-rehearsed scenes are daily gone through by the complaining sick. Thus, a white tongue, too white to suggest anything but the lime-washed wall of the prison cell, is frequently obtruded before the eyes of the surgeon, to whose stony heart it is expected to strike pity as evidence of hosts of dyspeptic troubles. When the tip of the nose, too, bears a corresponding snowy patch, the wo-begone air becomes especially ludicrous.

Again, an irregular and shaky pulse, which would be deemed an uncomfortable symptom elsewhere, here only suggests that the elbows have been drummed against the cell wall a few moments before.

Diarrhœa is a common cause of complaint, and we can imagine that the stirabout supplied with No. 1 diet may occasionally have laxative properties. If it does not yield to a dose of castor oil, followed by a chalk draught, the insistance on the use of the cell closet will generally show that the motions are nothing more serious than a carefully-prepared emulsion which need not be further described. The removal of the soap from the cell will sometimes check genuine relaxation of the bowels.

Hæmoptysis is a very common form of complaint, and is often a source of perplexity to the medical officer. We all know the mixture of frothy saliva—a blood produced by pricking and sucking the gums—too well to be taken in by it. But when the hæmorrhage is profuse, and when the thermometer and stethoscope give negative results, the cases occasionally are difficult. Here careful watching and frequent examination of the prisoner's clothes will sometimes bring to light packets of powdered glass or iron filings, which throw a flood of light on the diagnosis.

Toothache, again, is a complaint on which no amount of acumen on the part of the medical officer can decide on its genuineness. Extraction, however, is a prompt and crucial test which can readily be applied, and if, as I believe to be the case, prisoners will occasionally sacrifice a tooth for the sake of the slight change in the monotony of prison life that a visit to the surgery affords, we must, in consideration of the excellent practice it offers, forgive the deceit.

Fits are not as popular among malingerers as might be supposed. They require considerable knowledge and ingenuity, and no small amount of good acting, to meet with any measure of success. Since I began this paper I saw an account of an accomplished mimic who has at length got into trouble. His stock-in-trade consisted of a piece of soap with which to produce the foaming at the mouth, which is so convincing to the lay mind. Epilepsy is occasionally feigned by prisoners in order to get into association with other prisoners similarly affected, and generally to have an easy time. The following case is copied verbatim from the notes of Dr. Quinton: "A prisoner who was already in the hospital, and who had regurgitant disease of the mitral valve was reported in the night to be suffering from a fit. Remembering his cardiac mischief, I had visions of genuine cerebral symptoms from embolism in my head when I went to his bedside. He lay on his back in bed with his limbs relaxed, breathing slowly and stertorously, and apparently unconscious. When his eyelids were raised, both eyeballs were seen to be turned up, and to the right side. Pinching his legs produced no visible effect, but reflex action was excited by tickling the soles. No pulsation could be felt at either wrist or on the brachials, but the heart's action was vigorous, and the systolic apex beat was loud and distinct. On cutting open his shirt to examine higher up the arms I found in each axilla a pad which consisted of a four-ounce loaf wrapped round with rags. By firmly compressing these against the chest-wall he had succeeded in completely obliterating the pulses. The discovery of his trick had the instantaneous effect of restoring all his faculties.

"This prisoner had evidently seen and studied much, and yet there was no obvious motive for his fraud. There was little doubt, however, that it was done through *esprit de corps*, with a view of feeling the pulse of the new doctor."

Obscure general pains are frequently complained of, and imposition is difficult to detect unless the malingerer tries to prove too much, in which case he generally, overacts his part. The etiology of these pains is generally, according to the patient, old "special" taint. "I feel every change in the weather," said such a one, "for I am as full of mercury as a walking barometer."

*(To be continued.)*

